# Weighted quantile sum (WQS) mixed-effects model

**DOI:** 10.1016/j.mex.2025.103580

**Published:** 2025-08-22

**Authors:** Chris Gennings, Vishal Midya, Stefano Renzetti, Nicholas DeFelice

**Affiliations:** a Icahn School of Medicine at Mount Sinai, NY, NY, USA; b University of Parma, Parma, Italy

**Keywords:** Repeated measures, Environmental exposures, Mixture effect, Intra-subject correlated outcomes

## Abstract

Human-relevant environmental exposures typically have complex correlation patterns and may result in a mixture effect. That is, individual exposures may be below an effect level, however, the joint action (e.g., additive toxicity) of the components may produce significant effects. Weighted Quantile Sum (WQS) Regression has been used to estimate mixture effects using an empirically weighted index associated with an outcome of interest. The current WQS regression methodology assumes independence across subjects and does not permit multiple intra-subject outcome measures, which is a research gap. We extend WQS regression to the case when the data include multiple outcome variables or repeated measures with intra-subject correlation.

• Data are randomly split and the weights for the weighted index are estimated with resampling in the training set.
• Inference is conducted in repeated holdout validation sets to improve the stability of WQS estimates.
• A mixed-effects model is used in the holdout datasets to accommodate intra-subject correlated outcome variables for statistically valid inference relating the weighted index variables to the outcome(s). The new WQS mixed-effects model was applied to a pilot study of the impact of environmental conditions on kidney function with potential sex-specific differences in long-distance runners with repeated 20 km runs.

## Graphical abstract

**Figure:**
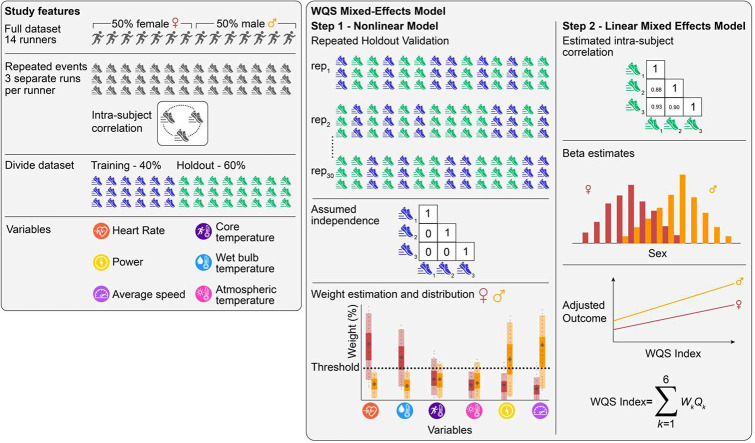

## Specifications table

**Table:** 

**Subject area**	Environmental Science
**More specific subject area**	Environmental epidemiology
**Name of your method**	WQS mixed-effects model
**Name and references of original method**	Carrico C, Gennings C, Wheeler DC, Factor-Litvak P. Characterization of Weighted Quantile Sum Regression for Highly Correlated Data in a Risk Analysis Setting. J Agric Biol Environ Stat. 2015;20(1):100–20. Epub 20,141,224. doi: 10.1007/s13253–014–0180–3. PubMed PMID: 30,505,142; PMCID: PMC6261506. Tanner EM, Bornehag CG, Gennings C. Repeated holdout validation for weighted quantile sum regression. MethodsX. 2019;6:2855–60. Epub 20,191,122. doi: 10.1016/j.mex.2019.11.008. PubMed PMID: 31,871,919; PMCID: PMC6911906.
**Resource availability**	The gWQS R package for conducting a repeated holdout validation for WQS regression is available on CRAN. R code for the extension to the WQS mixed-effects model is available in the supplementary material and will be available in the next version of the gWQS R package.

## Background

Weighted Quantile Sum (WQS) regression has been used in analyses of environmental health data of exposures with complex correlation patterns where the empirically weighted index represents the mixture effect of the components related to the outcome [[1], [2], [3], [4]]. A mixture effect addresses the potential joint action of the components which may each be below a level of concern for the individual component. In regression models of observational study data the beta coefficients of single components may not be significant but are in the direction of the hypothesis of interest. Focusing inference on the joint action of these components is more powerful to detect a significant signal, i.e., the mixture effect, using a single degree-of-freedom test. Other methods (e.g., Bayesian Kernal Machine Regression[5]) maintain the dimensionality of the number of components in estimating a response surface associated where the characterization of the important chemicals increases in complexity as the number of components increases. Further, shrinkage methods [6,7] attempt to identify important components which may be “arbitrary” representatives[6] from correlated groupings and may thereby eliminate chemicals of concern. By reducing dimensionality to a weighted index as in WQS regression, the number of components in the weighted index may be large and even larger than the sample size [3,4]. Finally, an ensemble step is an important feature of WQS regression as it accommodates complex correlation patterns among the components through resampling. Methods such as q g-comp[8] simultaneously estimate beta coefficients for each component without an ensemble method and may therefore suffer from the reversal paradox[1,9,10].

The strategy includes ensemble steps to construct the empirically-weighted index based on resampling. The weights are interpretable as the components are transformed to the same units using quantile scoring. Beta coefficients associated with the WQS index are estimated using repeated holdout analyses where the median, 2.5 % and 97.5 % are evaluated. In general, the approach can be used to construct a weighted index of components that make sense in a unidimensional construct. WQS regression has been used, for example, to provide insight into the association between prenatal exposures and neurodevelopmental and metabolic changes during childhood[[11], [12], [13], [14]] and the associations between exposures and the microbiome [15] and nutrition[16].

WQS regression typically involves independent observations across subjects. When study designs include multiple observations per subject, an extension of WQS regression is warranted. Examples include (i) measures of environmental exposures and multiple neurodevelopmental scales per subject; (ii) prenatal exposures and multiple health domains measured in childhood (neurodevelopment, immune function, metabolism, and growth), and (iii) repeated events (conditions and an outcome) measured per subject. The case study evaluated herein includes data from multiple runners evaluated at three separate runs. The components measured per subject during each run include speed, endurance measures, internal measures of fitness (i.e., heart rate and breathing rate)and external measures (heat, humidity). Kidney function biomarkers were evaluated before and after each run.

We propose an extension of WQS regression that includes a random effect in the evaluation of the multiple repeated holdout datasets to form a novel extension for WQS mixed-effects regression. The approach incorporates multiple intra-subject outcomes that may be biologically/physiologically related, thereby providing more information for the analysis of the mixture effect of the components.

## Method details

**Fig. 1:**
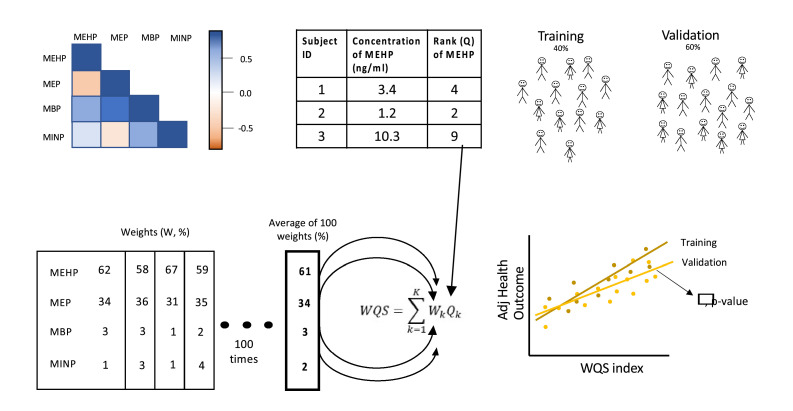
Weighted Quantile Sum regression is a model designed to evaluate the mixture effect of components with complex correlation patterns. Based on the measured concentration levels from each participant, the concentrations are first ranked into quantiles (here, deciles). The model splits the data into training (40 %) and validation (60 %) sets. In the training set, the mean weight of each chemical is estimated across 100 bootstrap samples. A weighted index, WQS, is then derived by multiplying the mean weight by the quantile of each component per subject. The higher the weights the higher the contribution to the weighted index. The weighted index can be derived in a positive or negative direction associated with the outcome of interest. Finally, the weighted index is tested in the validation set of the data in a generalized linear model.

We have developed a novel mixed-effects WQS regression model and we demonstrate it herein using the WQS stratified interaction regression model which results in empirically estimated intra-strata weighted indices and beta coefficients. As shown in the schematic in Fig. 1, the first step in WQS regression is to quantile score the values of the components to be used in the weighted index and then to randomly split the data into training and holdout sets. We generally use 40 % of the data in the training set and 60 % in the holdout set with the increased sample size in the step involving hypothesis testing. Using nonlinear regression, in the training set, we estimate the weights for each component in a weighted quantile (e.g., decile-scored components) sum, averaged across either bootstrap samples or random subsets of components. In the schematic, component weights are estimated from 100 bootstrap samples. We generally split the data multiple times to obtain repeated holdout datasets to improve generalizability[2] (graphical abstract step 1).

The first step focuses on the estimation of weights and not hypothesis testing, so a misspecified covariance structure is of less concern. Following the logic of nonlinear estimation in a quasi-likelihood framework and GEE modeling for large samples, assuming the model is correctly specified, the mean model parameters are accurate even when the covariance assumption is incorrect, here assuming independence [17,18]. This step is illustrated in the graphical abstract step 1 where the assumed intra-subject covariance structure is that of independence.

In a WQS stratified-interaction model, we estimate strata-specific weights and beta coefficients using a nonlinear estimation algorithm. For two strata (e.g., sex) and c components in the mixture, there are 2c weights to be empirically estimated. Typically, for two strata, we estimate the slope for the reference group (say, $b_{1}$) and an interaction parameter for the difference in slopes between the two groups (say, $b_{12}$). The slope for the other strata (other than the reference group) is $b_{2}\;=\;b_{1}\;+\;b_{12}$. (graphical abstract, bottom figure in middle column) When the number of parameters is large relative to the sample size, we use the random subset ensemble step instead of bootstrap sampling.

The second step in WQS regression is to test for the significance of the beta coefficient(s) in the holdout dataset, conditioning on the estimated weights from the first step. To improve generalizability, we conduct a repeated holdout strategy using many (e.g., rh=30or more) splits and holdout datasets. We augment the standard WQS model with the repeated holdouts using a generalized linear mixed-effects model that accommodates the intra-subject correlated strata-specific scores. (graphical abstract, 2nd step, right panel) All models are adjusted by covariates and confounders. The *gwqs* R package is thus augmented with the *nlme* package, which adds random effects to the linear model with the constructed weighted index and covariates. The following outline sets further details.

A. Randomly split the data (N observations with c components for the mixture) into a training (e.g., 40 %) and holdout (60 %) set with complete data for covariates and mixture components.
B. *Ensemble step*: Use a nonlinear regression model to estimate weights and beta coefficients, adjusted by covariates. Repeat many times by either resampling the observations in a bootstrap sample (e.g., b=100) or randomly selecting subsets of the c components (e.g., $\sqrt{c}$) a large number of times (e.g., b=1000) and average across the resulting weight estimates to construct the final weights and corresponding WQS index.
C. *Repeated holdout step:* Repeat steps A and B many times (e.g., rh=100) and evaluate the distribution of weights. The gWQS R package creates a vector called *vindex* which denotes the row numbers of the dataset included in each repeated holdout dataset. Merge the ID variable with each of the rh datasets denoted as lists.
D. *Mixed-effects estimation*: Estimate a mixed-effects model using each of the holdout datasets specified in the *vindex* in the gWQS package parameterized to include the wqs, strata, interaction terms, and covariates. For each analysis, specify the random-effect model using the ID. Create the estimated intra-subject correlation matrix. Summarize the resulting beta coefficients and working correlation matrices from the mixed-effects model.

As noted, the first step of our method focuses on the estimation of weights in the ensemble step and not hypothesis testing. The nonlinear estimation is conducted under the assumption of independence, and for large samples, the mean parameters are consistent, assuming the model is correct. A sensitivity analysis can be performed similar to Penalized GEE (PGEE)[19] using a lasso-type penalty term. Simulation studies of PGEE vs GEE indicate the former improves estimation accuracy over unpenalized GEE when the number of covariates in the model is the same order of magnitude as the sample size. An important feature of PGEE is that the consistency of model selection holds even if the working correlatin structure is misspecified.

The mixed-effects estimation step conditions on the estimated weights, and their average, from the training datasets to conduct the repeated application of a mixed-effects model in the repeated holdout datasets. Standard WQS regression also conditions on the weights from the training set, but herein, we also assume estimation of the weights under the potentially incorrect assumption of independence and assume the weights are at least approximately accurate.

## Method illustration

Our example dataset is from a pilot study of the impact of environmental conditions on runners. As such, the results are a demonstration of the method and require validation with a larger sample size for generalizable conclusions.

Study participants (*N* = 14, 7 men and 7 women) were evaluated during 3 separate long-distance runs in the summer of 2023 in New York City, starting and ending in Central Park. Runners were strapped with monitors of exposures (heat, humidity), speed and power, and biomonitoring of heart rate and core body temperature. Outcome measures included pre and post-measures of kidney function. The running route was broken into 5 regions (Central Park, Harlem, Washington Heights, West Side Highway, and Central Park).

In an initial analysis, we considered percent change in creatinine from pre- and post-race measures (i.e., (post-pre)/pre). Models were adjusted by sex and age. With 30 components (average speed, power, heart rate, core temperature, wet bulb temperature, atmospheric temperature within each of the 5 route regions) we used a random subset ensemble step (200 random sets of size 10) and 30 repeated holdouts. As we anticipated differences between men and women, we conducted a WQS stratified interaction model that allows for different weights and beta coefficients per sex. The final step is to estimate a mixed-effects model with stratified interaction parameterization. To conduct the ensemble step of the proposed method, we inflate the sample size by the number of intra-subject outcome variables (here, the number of runs per subject, i.e., from 14 subjects to 42=14 × 3) by constructing the data structure into a long format. And in the stratified interaction model, we also increase the number of parameters (component weights) by the number of strata (i.e., 30 components to 60), thereby allowing strata-specific weights. We used the random subset ensemble step as the number of parameters exceeded the sample size. We used random subsets of size 10 which is roughly the number of strata times the square root of the number of components. We pulled 200 random subsets per repeated holdout analysis. The boxplots for the weights indicated non-zero estimates for all repeated analyses providing evidence that the number of random subsets was adequate to sample from all parameters. We used 30 repeated holdouts – a minimum number which seemed adequate for a pilot study.

With 30 repeated holdouts, we evaluate the distribution of the estimated beta coefficients where $b_{1}$ is the beta for the reference group (females). The interaction term, $b_{12}$, is the difference in the wqs slopes between sexes. Using the distribution of the interaction terms (e.g., $b_{12}$), the WQS slope for the males is $b_{2}=b_{1}+b_{12}$. The interaction is not significant (Table 1), indicating there is no significant difference in the slopes between men and women relating the WQS index with percent change in creatinine. The slopes are both positive and borderline significant in females (median $b_{1}=0.104$) and males (median $b_{2}=0.196$) (Table 1). However, the median slope for males was roughly twice that of females (Table 1). The estimated intra-subject correlations between runs were all above 0.85 (Table 1). Interestingly, even though the slopes are not significantly different between females and males, the average estimated weights are different (Fig. 1). Creatinine changes in females are most associated with late-race heart rate (R4 and R5) and humidity (i.e., wet bulb temperature). In contrast, changes in males are most associated with heart rate in race region R1, and early (R1 and R2) and late (R5) measures of speed and power.

**Table 1:** Results from WQS stratified interaction mixed-effects model of **percent change in creatinine** using a random subset ensemble step with 200 random subsets of size 10 where components are decile scaled. The slopes are medians across 30 repeated holdouts from the linear mixed effects models.

Coefficient	Median		95 % CI from linear mixed model	
WQS: Female	0.104		(−0.008, 0.203)	
WQS: Male	0.196		(−0.074, 0.305)	
WQS*Sex Interaction	0.098		(−0.174, 0.263)	
Gender: M	−0.027		(−0.269, 0.398)	
Age (scaled)	0.046		(0.007, 0.087)	
Median Intra-subject Correlation Estimates	R01	R02		R03
R01	1			
R02	0.88	1		
R03	0.93	0.93		1

Finally, the adjusted models indicated that older runners had significantly increased changes in creatinine.

Finding differences between men and women runners is as anticipated due to physiological/biological differences in muscular strength and aerobic capacity [20].

## Limitations and future work

In studies with small numbers in some strata, a random split of the data may not have all strata represented. To address this issue, we force random splits to follow the same proportions as observed in the full dataset using the *createDataPartition()* function in R. In small to moderate-sized studies with a large number of components, the use of the random subset version of the ensemble step is likely to randomly select components that are not balanced across the strata. In extreme random subset cases, there may not be data from each strata. The random subset ensemble step samples from the pool of variables in the model, even in a stratified interaction model. We will change this in stratified models in the next version of the gwqs package to instead sample the original variables and impose the stratified structure, thereby guaranteeing estimates for each strata in each sampled analysis.

The estimation of the intra-subject correlation structure is based on empirical correlation estimates from the random effects. More parsimonious structures such as AR(1) and Compound Symmetry are not yet included.

**Fig. 2:**
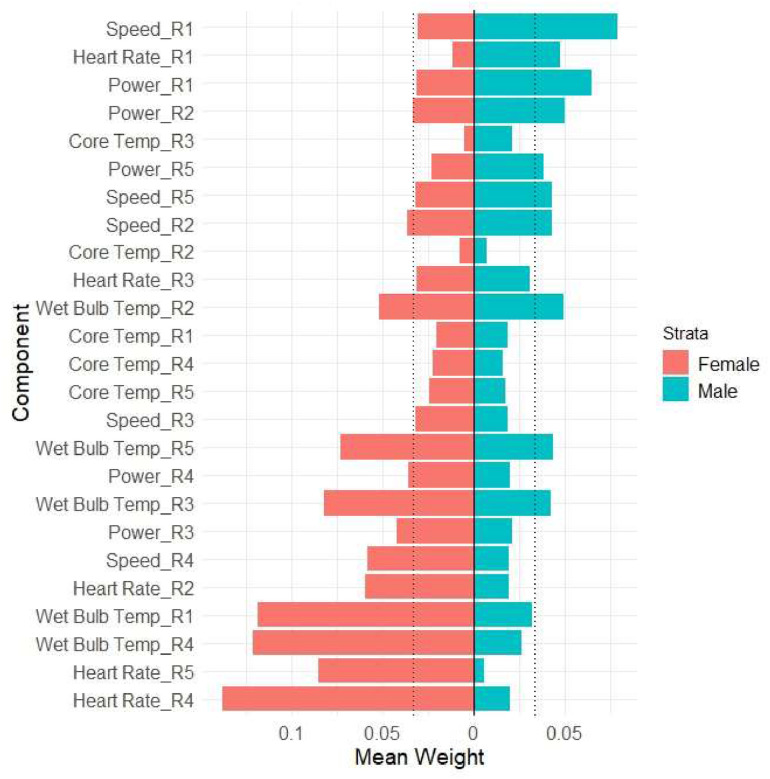
Divergent plot of average weights across 10 repeated holdouts in the WQS stratified interaction model associated with percent change in creatinine.

The sample size is small in our case study and the number of parameters is large relative to n. We have conducted a sensitivity analysis using a PGEE-type analysis[19] with a lasso-type penalty term in the estimation step. The results are similar to those in Table 1 and Fig. 2 and are presented as supplemental data (Table S1 and Figure S1).

The WQS mixed-effects model provides important flexibility for analyses of environmental health cohort studies. For example, we anticipate its use in cohort data of developmental studies with prenatal exposures to mixtures following the Developmental Origins of Health and Disease (DOHaD) theory where developmental outcome measures over time are of importance. The method is also applicable for neurodevelopmental studies where a battery of neuroscales are measured. Alternative approaches include the Bayesian varying coefficient kernal machine regression model[21] and BKMR, both of which permit the estimation of random effects, but neither allow for interaction with categorical covariates. On the other hand, q g-comp allows for an interaction term with a categorical covariate and accounts for repeated measures through a GEE implementation[22]. But q g-comp conducts the simultaneous estimation of beta coefficients for all components and thereby suffers from multicollinearity and reversal paradox concerns.

## Conclusions

We have extended WQS regression to the case when the data include multiple outcome variables or repeated measures with intra-subject correlation. The repeated holdout validation is used to provide distributions of estimated weights for the empirically weighted indices and beta coefficients. The approach allows the incorporation of strata-specific weights as demonstrated in the case study. Inference in the final step is based on mixed-effects linear models using average weighted indices from the first step. The WQS mixed effects model is a useful extension of WQS regression, allowing for study data with multiple intra-subject measures.

## Ethics statements

Relevant informed consent was obtained from the subjects in the P30 pilot study (#STUDY-21–01,725).

## Related research article

Carrico C, Gennings C, Wheeler DC, Factor-Litvak P. 2015. Characterization of Weighted Quantile Sum Regression for Highly Correlated Data in a Risk Analysis Setting. J Agric Biol Environ Stat 20:100–120; doi:10.1007/s13253–014–0180–3.

## CRediT authorship contribution statement

**Chris Gennings:** Conceptualization, Methodology, Software, Formal analysis, Writing – original draft. **Vishal Midya:** Validation, Writing – review & editing. **Stefano Renzetti:** Writing – review & editing. **Nicholas DeFelice:** Investigation, Funding acquisition, Writing – review & editing.

## Declaration of competing interest

The authors declare that they have no known competing financial interests or personal relationships that could have appeared to influence the work reported in this paper.

## Data Availability

Data will be made available on request.

## Appendix Supplementary materials

Supplementary material *and/or* additional information [OPTIONAL] Image, application 1
