# Supplementary material for: Weighted quantile sum (WQS) mixed-effects model
Source: MethodsX. 2025 Aug 22;15:103580. doi: 10.1016/j.mex.2025.103580 (PMC12450740; doi:10.1016/j.mex.2025.103580)
Supplement: Supplementary file 1 — Supplementary material and/or additional information [OPTIONAL] [file mmc1.docx]

**Supplemental Data**

**Table S1:** Results from WQS stratified interaction mixed-effects model of **percent change in creatinine** using a random subset ensemble step with 200 random subsets of size 10 where components are decile scaled using a lasso-type penalty term in the estimation step. The slopes are medians across 30 repeated holdouts from the linear mixed effects models.

| **Coefficient** | **Median** | **95% CI from**  **linear mixed model** |
| --- | --- | --- |
| WQS: Female | 0.104 | (-0.017, 0.232) |
| WQS: Male | 0.151 | (-0.087, 0.345) |
| WQS*Sex Interaction | 0.068 | (-0.215, 0.272) |
| Gender: M | -0.045 | (-0.392, 0.432 |
| Age (scaled) | 0.036 | (0.001, 0.086) |

| Median Intra-subject Correlation Estimates | R01 | R02 | R03 |
| --- | --- | --- | --- |
| R01 | 1 |  |  |
| R02 | 0.88 | 1 |  |
| R03 | 0.91 | 0.95 | 1 |

**Figure S1:** Divergent plot of average weights across 10 repeated holdouts in the WQS stratified interaction model associated with percent change in creatinine using a lasso-type penalty in the estimation step.


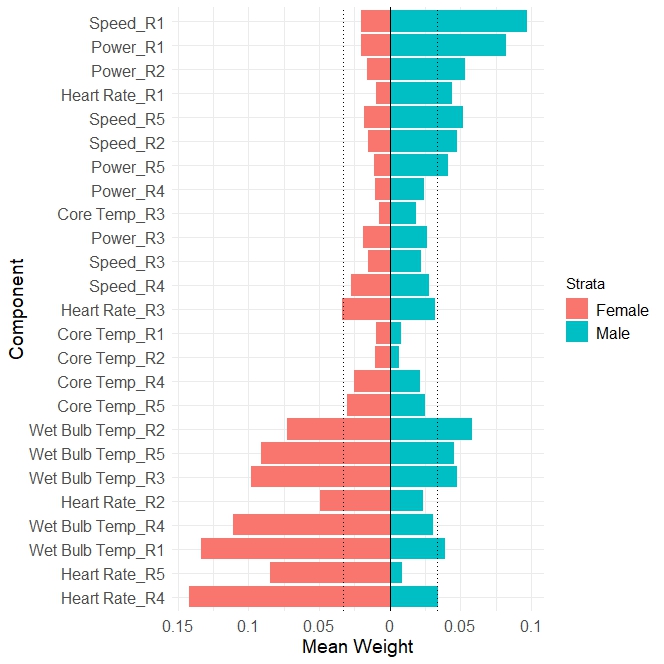


**R code for analysis of the running study**

# Running study pilot analysis, July 2025

#rm(list = ls())

#install.packages("gWQS")

library(gWQS)

library(broom)

library(tidyr)

library(tidyverse)

library(dplyr)

library(nlme)

library(zoo) # for imputing

# define the path

#directory_path = "/Users/gennic01/Desktop/TEMP RESEARCH/Nicolas DeFelice/running study pilot/"

directory_path = "C://Users/gennic01/Desktop/RESEARCH/Nick deFelice/"

# import the dataset

#dataset = read.csv(paste0(directory_path, "run_section_summaries_w_labs_2024-03-29.csv"))

dataset = read.csv(paste0(directory_path, "Running data/run_section_summaries_w_labs_2024-12-06.csv"))

dim(dataset)

#dataset = dataset[complete.cases(dataset),]

dim(dataset)

names(dataset)

hist(dataset$run_pct_chg_creatinine, xlab="Crt %change", col="blue")

hist(dataset$Age, xlab="Age", col="blue")

# define the chemicals to include in the mixture

mixture=names(dataset)[grep("avg",names(dataset))]

mixture

mixturePM = names(dataset)[grep("pm25", names(dataset))]

mixturePM

mixture=mixture[-grep("_R0",mixture)]

mixture=mixture[-grep("hexoskin",mixture)]

mixture

mixture = c(mixture, mixturePM)

mixture

################################################################

################################################################

# correlation among the chemicals

#install.packages("corrplot")

library("corrplot")

dataset_ = dataset[complete.cases(dataset[,mixture]),]

dim(dataset_)

forcorr = data.frame(dataset_[,mixture])

corrplot(cor(forcorr), method="circle", sig.level = c(.001, .01, .05), type="upper")

###############################################################

############################# single chemical analyses

### log2 transformed

datalog = dataset

datalog[, mixture] <- lapply(datalog[, mixture], log, 2)

## Quantile-transformation

dataquant = dataset

dataquant[, mixture] <- lapply(dataquant[, mixture], ntile, 4)

#######################################################

# Linear Model w/ one biomarker

# create a list of markers of interest

biomarkers <- mixture

# linear model

lm_fit <-

lapply(biomarkers, function(x){

temp = dataquant[,c(mixture, "run_pct_chg_creatinine",

"Age", "Gender")]

temp= temp[complete.cases(temp),]

glm(

formula = substitute(run_pct_chg_creatinine ~ i + Age + as.factor(Gender),

list(i = as.name(x))),

data = temp,

family = gaussian())

})

# creates a table with multiple lists

# number of lists created = number of biomarkers chosen

# extract coefficients with confidence interval

lm_coefs <- lapply(lm_fit, tidy, conf.int = T)

names(lm_coefs) <- biomarkers

# combine all lists into one big table

# gives estimates and CI for biomarkers and variables

lm_coefs <- bind_rows(lm_coefs, .id = "biomarkers")

# subset estimates and CI for _only the biomarkers_

lm_coefs <- filter(lm_coefs, term %in% biomarkers)

warnings()

lm_coefs

directory_path_out = "C://Users/gennic01/Desktop/RESEARCH/Nick deFelice/"

#write.csv(lm_coefs, file=paste0(directory_path_out, "single_log2.csv"))

write.csv(lm_coefs, file=paste0(directory_path_out, "single_quartile.csv"))

####################################################################################

# covariate only model

cov_only = glm(run_pct_chg_creatinine ~ as.factor(Gender)+Age, data = dataset)

summary(cov_only)

#####################################################################################

# WQS regression

#####################################################################################

results1 = gwqs(run_pct_chg_creatinine ~ wqs, mix_name = mixture, data = dataset, q = 4, validation = 0.6,

b = 100, b_pos = TRUE, b_constr = FALSE, family = "gaussian", seed = 123,

plan_strategy = "multisession")

gwqs_barplot(results1)

gwqs_scatterplot(results1)

gwqs_fitted_vs_resid(results1)

gwqs_summary_tab(results1)

sum(results1$bres$b1 < 0)

## all 100 bootstraps had positive beta estimates in the GLM

summary(results1$fit)

results1$final_weights

# adjusting for covariates:

# positive direction - when adjusted the association is in the positive direction

result2 = gwqs(run_pct_chg_creatinine ~ wqs + Gender + scale(Age),

mix_name = mixture, data = dataset, q = 4,

validation = 0.6, b = 100, b_pos = TRUE, b_constr = TRUE, family = "gaussian",

seed = 123, plan_strategy = "multisession")

gwqs_barplot(result2)

gwqs_summary_tab(result2)

gwqs_scatterplot(result2)

sum(result2$bres$b1 > 0) ## 100 are positive beta estimates

#compare to random subset WQS

result4 = gwqs(run_pct_chg_creatinine ~ wqs + Gender + scale(Age),

mix_name = mixture, data = dataset, q = 10,

validation = 0.6, b = 500, b_pos = TRUE, b_constr = TRUE, family = "gaussian",

seed = 123, rs=TRUE, n_var=7, plan_strategy = "multisession")

gwqs_summary_tab(result4)

gwqs_barplot(result4)

gwqs_scatterplot(result4)

gwqs_weights_tab(result4)

sum(result4$bres$b1 > 0)

# run the wqs model using the stratified interaction model

dataset$Gender = as.factor(dataset$Gender)

table(dataset$Gender)

# run the wqs model using the stratified variables in the mixtures with interaction

result6 = gwqs(run_pct_chg_creatinine ~ wqs*Gender +

scale(Age),

stratified="Gender", mix_name = mixture, data = dataset, q = 4,

validation = 0.6, b = 500, rs=TRUE, n_vars = 10,

b1_pos = TRUE, b_constr = TRUE, bint_cat_pos=TRUE, family = "gaussian",

seed = 123)

gwqs_summary_tab(result6)

gwqs_scatterplot(result6)

gwqs_fitted_vs_resid(result6)

gwqs_barplot(result6)

#install.packages("caret")

library(caret)

set.seed(123)

vr <- createDataPartition(dataset$Gender, p = 0.6, times = 1)

vr <- lapply(vr, function(i) 1:nrow(dataset) %in% i)

dataset$Gender = as.factor(dataset$Gender)

result6 = gwqs(run_pct_chg_creatinine ~ wqs*Gender +

scale(Age),

stratified="Gender", mix_name = mixture, data = dataset, q = 4,

validation = 0.5, b = 200, rs=TRUE, n_vars = 10,

b1_pos = TRUE, b_constr = TRUE, bint_cat_pos=TRUE, family = "gaussian",

seed = 123, validation_rows = vr)

gwqs_summary_tab(result6)

gwqs_scatterplot(result6)

gwqs_fitted_vs_resid(result6)

gwqs_barplot(result6)

summary(result6)

#compare to result6 but here use lambda penalty similar to PGEE for small sample sizes

result7 = gwqs(run_pct_chg_creatinine ~ wqs*Gender +

scale(Age),

stratified="Gender", mix_name = mixture, data = dataset, q = 4,

validation = 0.5, b = 200, rs=TRUE, n_vars = 10,

b1_pos = TRUE, b_constr = TRUE, bint_cat_pos=TRUE, family = "gaussian",

lambda=10,

seed = 123, validation_rows = vr)

gwqs_summary_tab(result7)

gwqs_barplot(result7)

#####################################################################

## divergent plots

## load libraries

library(gapminder)

library(tidyverse)

library(stringr)

forplot = result6rh$final_weights

#forplot = result7rh$final_weights ## specify the data to be used in the plot; i.e., WQS str or WQS str int model

forplot$mean_weight = forplot$Estimate

sum(forplot$Estimate)

forplot$strata = str_sub(forplot$mix_name,-1)

forplot$chem = str_sub(forplot$mix_name,5,nchar(as.character(forplot$mix_name))-2)

forplot$region = str_sub(forplot$chem,-3)

forplot = forplot %>% mutate(chem2 = ifelse(str_sub(chem,1,4)=="cale", paste0("Heart Rate", str_sub(chem,-3)),

ifelse(str_sub(chem,7,11)=="speed", paste0("Speed", str_sub(chem,-3)),

ifelse(str_sub(chem,7,11)=="power", paste0("Power", str_sub(chem,-3)),

ifelse(str_sub(chem,1,4)=="upas", paste0("Wet Bulb Temp", str_sub(chem,-3)),

ifelse(str_sub(chem,1,4)=="core", paste0("Core Temp", str_sub(chem,-3)),

ifelse(str_sub(chem,1,4)=="pm25", paste0("PM2.5", str_sub(chem,-3)),

chem))))))

)

forplot$strataname = ifelse(forplot$strata == "M", "Male", "Female")

names(forplot)

resultstr <- forplot %>%

mutate(mean_weight = ifelse(strata == "M",

mean_weight,

-1*mean_weight))

resultstr

dim(resultstr)

## calculate breaks values

breaks_values <- pretty(resultstr$mean_weight)

ggplot(data=resultstr,

aes(reorder(x = chem2, mean_weight), y = mean_weight, fill = strataname))+

geom_bar(stat = "identity")+

coord_flip()+

scale_y_continuous(breaks = breaks_values,

labels = abs(breaks_values))+

theme_minimal()+

theme(axis.text=element_text(size=12),

axis.title=element_text(size=14),

legend.text = element_text(size=12))+

geom_hline(yintercept = 2*1/nrow(resultstr), linetype='dotted', col='black')+

geom_hline(yintercept = 2*(-1/nrow(resultstr)), linetype='dotted', col='black')+

geom_hline(yintercept = 0, col='black')+

labs(x="Component", y="Mean Weight", fill="Strata")

wt_perc_males = round(100*sum(resultstr[resultstr$strata == 'M',]$mean_weight)/sum(forplot$Estimate),1)

wt_perc_males

##########################################################

##########################################################

#repeated holdout WQS

##########################################################

##########################################################

table(dataset_impute$Gender)

#install.packages("caret")

library(caret)

set.seed(123)

vr <- createDataPartition(dataset$Gender, p = 0.5, times = 30)

vr <- lapply(vr, function(i) 1:nrow(dataset) %in% i)

dim(dataset)

mixture # error message: summary(i$mfit$m_f)$coefficients[intpos, "Estimate"] : (subscript) logical subscript too long

# so reduced the components in the mixture - took out pm

#mixture=mixture[-grep("pm25",mixture)]

mixture

result6rh = gwqs(run_pct_chg_creatinine ~ wqs*Gender +

scale(Age),

stratified="Gender", mix_name = mixture, data = dataset, q = 4,

validation = 0.5, b = 200, b1_pos = TRUE, b_constr = TRUE, bint_cat_pos=TRUE,

family = "gaussian", seed=123,

rh=30, rs=TRUE, n_vars=10,

validation_rows = vr,

plan_strategy = "multisession")

gwqs_summary_tab(result6rh)

gwqs_summary_tab(result6rh, sumtype = "perc")

gwqs_boxplot(result6rh)

gwqs_weights_tab(result6rh)

summary(result6rh$fit)

result6rh$final_weights

result6rh$final_weights$mean_weight = result6rh$final_weights$Estimate

directory_path_out = "C://Users/gennic01/Desktop/RESEARCH/Nick deFelice/"

write.csv(result6rh$wmat, paste0(directory_path_out, "stintWQSwtsrh30.csv"))

# with penalty term due to small sample size - similar to PGEE

result7rh = gwqs(run_pct_chg_creatinine ~ wqs*Gender +

scale(Age),

stratified="Gender", mix_name = mixture, data = dataset, q = 4,

validation = 0.5, b = 200, b1_pos = TRUE, b_constr = TRUE, bint_cat_pos=TRUE,

family = "gaussian", seed=123,

rh=30, rs=TRUE, n_vars=10,

lambda=10,

validation_rows = vr,

plan_strategy = "multisession")

gwqs_summary_tab(result7rh)

gwqs_summary_tab(result7rh, sumtype = "perc")

gwqs_boxplot(result7rh)

gwqs_weights_tab(result7rh)

summary(result7rh$fit)

result7rh$final_weights

result7rh$final_weights$mean_weight = result7rh$final_weights$Estimate

directory_path_out = "C://Users/gennic01/Desktop/RESEARCH/Nick deFelice/"

write.csv(result7rh$wmat, paste0(directory_path_out, "stintWQSwtsrh30_lambda.csv"))

##################################################################################

##################################################################################

# WQS mixed effects model from repeated holdout data

##################################################################################

##################################################################################

domaingWQSstintrh = result6rh

#domaingWQSstintrh = result7rh

summary(domaingWQSstintrh)

b1_gwqs = domaingWQSstintrh$coefmat[,2]

b12_gwqs= domaingWQSstintrh$coefmat[,5]

b2_gwqs = b1_gwqs +b12_gwqs

quantile(b1_gwqs, prob=c(0.025, 0.5, 0.975))

quantile(b2_gwqs, prob=c(0.025, 0.5, 0.975))

quantile(b12_gwqs, prob=c(0.025, 0.5, 0.975))

############################################

table(dataset$run_id, dataset$participant_num)

variables = c("Gender", "Age", "run_id")

head(dataset[,c("participant_num", variables)])

dataset$Y = dataset$run_pct_chg_creatinine

data.complete = dataset[complete.cases(dataset[c(mixture, variables, "Y")]), ]

dim(data.complete)

head(data.complete[,c("participant_num", variables)])

ID=data.complete$participant_num

run_id=data.complete$run_id

b1 <- NA_real_

b2 <- NA_real_

b12 <- NA_real_

bGenderM <- NA_real_

bscaleAge <- NA_real_

rho12 = NA_real_

rho13 = NA_real_

rho23 = NA_real_

rh = 30

for( i in 1:rh){

compcasedata = cbind(domaingWQSstintrh$gwqslist[[i]]$data, ID, run_id)

data_test = compcasedata[domaingWQSstintrh$gwqslist[[i]]$vindex,]

lr <- lme(run_pct_chg_creatinine ~ wqs*Gender +

scale(Age),

# data = data_test, random= ~ domain | ID)

data = data_test, random= ~1 | ID)

summary(lr)

b1 <- c(b1, lr$coefficients$fixed[2])

b12 <- c(b12, lr$coefficients$fixed[5])

b2 <- c(b2, lr$coefficients$fixed[2] + lr$coefficients$fixed[5])

bGenderM <- c(bGenderM,lr$coefficients$fixed[3] )

bscaleAge <- c(bscaleAge, lr$coefficients$fixed[4])

predrandeff = lr$fitted[,2]

randeff = cbind(predrandeff, data_test[,c("ID", "run_id")])

reffwide <- randeff %>% pivot_wider(names_from = run_id, values_from = predrandeff)

reffwide

domaincors <- cor(reffwide[,-1],use="pairwise.complete.obs")

rho12 = c(rho12, domaincors[1,2])

rho13 = c(rho13, domaincors[1,3])

rho23 = c(rho23, domaincors[2,3])

}

summary(lr)

table(compcasedata$run_id)

summary(rho12[-1])

summary(rho13[-1])

summary(rho23[-1])

sum(b1[-1]>0)/rh

sum(b2[-1]>0)/rh

sum(b12[-1]>0)/rh

sum(bGenderM[-1]>0)/rh

sum(bscaleAge[-1]>0)/rh

quantile(b1[-1], c(0.025, 0.05, 0.5, 0.95, 0.975))

quantile(b2[-1], c(0.025, 0.05, 0.5, 0.95, 0.975))

quantile(b12[-1], c(0.025, 0.05, 0.5, 0.95, 0.975))

quantile(bGenderM[-1], c(0.025, 0.05, 0.5, 0.95, 0.975))

quantile(bscaleAge[-1], c(0.025, 0.05, 0.5, 0.95, 0.975))
